# Genome-wide identification and functional analysis of cupin_1 domain-containing members involved in the responses to *Sclerotinia sclerotiorum* and abiotic stress in *Brassica napus*

**DOI:** 10.3389/fpls.2022.983786

**Published:** 2022-08-01

**Authors:** Yizhou He, Yan Li, Zetao Bai, Meili Xie, Rong Zuo, Jie Liu, Jing Xia, Xiaohui Cheng, Yueying Liu, Chaobo Tong, Yuanyuan Zhang, Shengyi Liu

**Affiliations:** ^1^The Key Laboratory of Biology and Genetic Improvement of Oil Crops, The Ministry of Agriculture and Rural Affairs of the PRC, Oil Crops Research Institute of the Chinese Academy of Agricultural Sciences, Wuhan, China; ^2^State Key Laboratory of Biocatalysis and Enzyme Engineering, School of Life Sciences, Hubei University, Wuhan, China; ^3^Hubei Collaborative Innovation Center for Green Transformation of Bio-Resources, School of Life Sciences, Hubei University, Wuhan, China

**Keywords:** cupin_1 domain, germin-like protein, *Sclerotinia sclerotiorum* resistance, abiotic stress, GWAS, *Brassica napus*

## Abstract

Cupin_1 domain-containing proteins (CDPs) are ubiquitously present in higher plants, which are known to play essential roles in various biological processes. In this study, we carried out genome-wide characterization and systematic investigation of the *CDP* genes in *Brassica napus.* A total of 96 BnCDPs, including 71 germin-like proteins (GLPs; proteins with a single cupin_1 domain) and 25 CDP bicupins (proteins with two cupin_1 domains), were identified and clustered into six distinct subfamilies (I–VI) based on the phylogenic analysis, gene structure and motif distribution. Further analysis indicated that whole-genome duplication (WGD) and segmental duplication are main contributors to the species-specific expansion of the *BnCDP* gene family, and all the duplicated genes subsequently underwent strong purification selection. The promoter region of *BnCDPs* showed enrichment of *cis*-regulatory elements associated with development, hormone and stress, as well as transcription factor binding sites, which validates the prediction that *BnCDPs* are widely involved in plant growth and biotic and abiotic stress responses. The *BnCDPs* in different subfamilies exhibited obvious differences in expression among 30 developmental tissues/stages of *B. napus*, implying that *BnCDP*s may be involved in tissue- and stage-specific developmental processes. Similar trends in expression of most *BnCDPs* were observed under *Sclerotinia sclerotiorum* inoculation and four abiotic stresses (dehydration, cold, ABA and salinity), particularly the *BnGLPs* in subfamily I and III with single cupin_1 domain, revealing that *BnCDPs* are of great importance in the environmental adaption of *B. napus*. We then performed a genome-wide association study (GWAS) of 274 *B. napus* core germplasms on *S. sclerotiorum* resistance and identified four significantly associated loci harboring five *BnGLPs*. The expression levels of two candidate genes, *BnGLP1.A08* and *BnGLP1.C08*, were significantly correlated with *S. sclerotiorum* resistance. Their functional responses to multiple stages of *S. sclerotiorum* inoculation and four abiotic stresses were further examined through qPCR. Overall, this study provides rich resources for research on the function and evolutionary playground of *CDP* genes.

## Introduction

Adverse environmental conditions including biotic and abiotic stresses pose serious threats to crop productivity in agriculture and food security (Zhu, 2016; Zhao et al., 2022). How plants adapt to adverse environments is a critical issue of biological studies and global agricultural production. It is critical to tune the expression of stress-responsive genes for resistance and adaptation to various biotic and abiotic stresses. The cupin_1 domain-containing protein (CDP) coding genes such as *GLP* members tend to be induced by pathogen attack and abiotic stress, and play important roles in response to a number of biotic and abiotic stresses to improve the development and environmental adaption of plants (Dunwell et al., 2004). Cupin superfamily proteins, which were named based on a conserved β-barrel fold, were first discovered using a conserved motif found within germin and germin-like proteins from higher plants (Dunwell et al., 2000, 2004). To date, the cupin superfamily has been considered as one of the most functionally diverse super-gene families in plants (Dunwell et al., 2004; Brunetti et al., 2022). This superfamily contains 69 gene families according to the Pfam database (accessed on 1st, June 2022), including the cupin_1 domain-containing family (Mistry et al., 2021). The cupin superfamily can be divided to monocupin (one single cupin domain), bicupin (a duplicated cupin structure) and multicupin (>two cupin domains; Dunwell et al., 2004). Germin and germin-like protein (GLP), which contain a single cupin_1 domain and belong to monocupin (Dunwell et al., 2000, 2004), have been widely deciphered in various plants, while the duplicated cupin_1 domain protein (CDP bicupin) has been rarely characterized. GLPs are defined by their sequence homology to germin, which was initially identified as a germination-specific marker in wheat embryos (Thompson and Lane, 1980; Dunwell et al., 2008; Rietz et al., 2012). Both germin and GLP display extremely high resistance to proteasome activity, heating, extreme pH, and detergents (Woo et al., 2000). Due to the conserved sequences and similarity in structural characteristics, it is difficult to classify GLPs and germins (Agarwal et al., 2009). In general, germins belong to a well-conserved homogeneous group and can be uniquely found within cereal plant species, including barley (*Hordeum*), maize (*Zea*), oat (*Avena*), rice (*Oryza*), rye (*Secale*) and wheat (*Triticum*) (Lane, 2002). In contrast, the GLP proteins have a wider taxonomic distribution and are generally present in other land plants besides cereals (Dunwell et al., 2008). Despite similarities in sequence among the members of GLPs, they have undergone significant functional diversification, and this family comprises numerous classes of important enzymes such as superoxide dismutase (SOD) that converts superoxide to H_2_O_2_ and O_2_ (Gucciardo et al., 2007; Guevara-Olvera et al., 2012), oxalate oxidase (OXO) that degrades oxalic acid to H_2_O_2_ and CO_2_ (Sakamoto et al., 2015), and polyphenol oxidase, dioxygenases, isomerases, epimerases, synthases and decarboxylases (Davidson et al., 2010; Cheng et al., 2014).

The *CDP* genes have different spatial and temporal expression characteristics during development in a variety of plants, and their expression also varies greatly among different tissues such as roots, stems, leaves, flowers, seeds and embryos and various developmental processes of the same plant (Lu et al., 2010; Wang et al., 2013; Li L. et al., 2016). Among the 69 identified cupin genes in soybean, 35 were found to be expressed in at least one tissue, and most of them displayed distinct tissue-specific expression patterns (Wang et al., 2014). Expression profiling of *GLPs* in rice and *Arabidopsis thaliana* has demonstrated that many of the members are only expressed in certain tissues or developmental stages (Li L. et al., 2016). For example, *OsGLP3-3* and *OsGLP8-2* are specifically expressed in developing seeds and *OsGLP8-14* is preferentially expressed in developing panicles in rice (Li L. et al., 2016). These tissue specifically expressed *GLPs* may have essential functions in plant growth and development. For example, *OsGLP2-1*, which is specifically expressed in seed scutellum, positively regulates the dormancy of developing seeds through the abscisic acid and gibberellic acid signaling pathways (Wang et al., 2020); while *OsGLP1* is predominantly expressed in green vegetative tissues and down-regulation of its expression in transgenic rice resulted in a semi-dwarfism phenotype (Banerjee and Maiti, 2010). *GbGLP2* is mainly expressed in elongating fiber at 10 days post anthesis and negatively regulates fiber elongation in cotton (Sun et al., 2020). All these studies have demonstrated that *GLPs* play various roles in many tissues and organs or certain vital developmental stages of plants.

The *CDP* genes have long been considered be associated with responses to various biotic and abiotic stresses in different plant species (Thompson et al., 1995; Liu et al., 2004; Zou et al., 2007; Dong et al., 2008). Some GLP members with inherent OXO or SOD enzymatic activity can confer tolerance to biotic stress by hyper-accumulation of H_2_O_2_ and enhancement of cross-link between cell wall components during pathogen infection (Banerjee et al., 2010; Gangadhar et al., 2021). Riezt et al. identified 14 *BnGLP* genes in *Brassica napus* and demonstrated that both *BnGLP3* and *BnGLP12* have SOD activity, whose early induction is involved in the oxidative burst, and play a pivotal role in defense against *Sclerotinia sclerotiorum* (Rietz et al., 2012). *GmGLP10* positively regulates the resistance to *S. sclerotiorum*, and transgenic tobacco overexpressing *GmGLP10* from soybean showed significantly enhanced tolerance to oxalate acid and *S. sclerotiorum* infection (Zhang et al., 2018). Similarly, *A. thaliana* plants expressing sunflower *HaGLP1* exhibited higher reactive oxygen species accumulation and resistance against *S. sclerotiorum* and *Rhizoctonia solani* (Beracochea et al., 2015). Overexpression in *A. thaliana* of a novel *GLP* gene *GhABP19* from *Gossypium hirsutum* resulted in enhanced resistance to *Verticillium dahliae* and *Fusarium oxysporum* infection through its SOD activity and activation of the Jasmonic acid (JA) pathway (Pei et al., 2019). Moreover, *GLPs* also widely participate in defense against some other fungal pathogens such as *Blumeria graminis* (Yuan et al., 2021), *Verticillium longisporum* (Knecht et al., 2010), *Magnaporthe oryzae* (Liu et al., 2016) and *Aspergillus flavus* (Wang et al., 2013), as well as responses to viruses, bacteria, and even insect herbivores (Dunwell et al., 2008). Apart from biotic stress, the *CDP* genes are also widely involved in defense against abiotic stress in plants. For instance, *A. thaliana* with ectopic overexpression of soybean *GmGLP7* exhibited obviously enhanced tolerance to drought, salt and oxidative stress, and was hypersensitive to exogenous ABA treatment (Li Y. et al., 2016). Similarly, *A. thaliana* overexpressing *AhGLP2* or *AhGLP3* from peanut showed higher tolerance to salt stress (Wang et al., 2013). Knockout of *OsGLP1* by CRISPR/Cas9 resulted in higher sensitivity of rice plants to UV-B, suggesting that *OsGLP1* is involved in the acclimation to UV-B radiation (He et al., 2021). In addition, overexpression of *StGLP* in potato plants increased the H_2_O_2_ level, triggered the scavenging signaling pathways of reactive oxygen species and induced the expression of heat stress-responsive genes to enhance the tolerance to heat stress (Gangadhar et al., 2021). Besides, the *GLP* genes are also responsive to other abiotic stresses such as drought (Anum et al., 2022), heavy metal (Cheng et al., 2018) and wound (Wang et al., 2013).

*Brassica napus* (2n = 4x = 38, AACC) is an important source of vegetable oil and stock feed in the world, which is cultivated in an area of more than 36 million hectares with an annual seed yield of 72 million tons (FAO STAT^1^). In actual production, the yield of *B. napus* is threatened by various biotic (insect pest and disease) and environmental/abiotic (salinity, acidity, alkalinity, drought, heat and water-logging) stresses (Liu et al., 2022). Therefore, it is urgent to explore genes with durable disease resistance and tolerance to diverse abiotic stresses in crop breeding. Considering the important roles of *CDP* genes in resistance and adaptation of plants to various biotic and abiotic stresses, it is reasonable to speculate that *CDPs* may also function in the environmental adaptation of *B. napus*. However, there has been no genome-wide identification and systematic investigation of the *CDP* family represented by monocupion *GLPs* in *B. napus* to date. In this study, we identified a total of 96 *CDP* genes (including 71 *BnGLPs* and 25 CDP *bicupins*) in the *B. napus* genome through genome-wide analysis. In addition, we comprehensively analyzed their evolutionary relationships, gene structures, conserved motifs, *cis*-regulatory elements and transcription factor binding sites (TFBSs). Expression profiling in 30 *B. napus* tissues/stages demonstrated that *BnCDPs* are involved in tissue- and stage-specific developmental processes. Expression analysis under biotic (*S. sclerotiorum* infection) and abiotic (dehydration, cold, ABA and salinity) stress treatments together with GWAS on *S. sclerotiorum* resistance demonstrated that two *BnGLPs* are commonly responsive to multiple biotic and abiotic stresses. The findings provide important insights into the role of *BnCDPs* in resistance to biotic and abiotic stresses and lay a foundation for future functional study of the *CDP* genes.

## Results

### Identification of *BnCDP* gene family members in *Brassica napus*

A total of 96 *BnCDP* genes with the cupin_1 domain were identified in *B. napus* through HMMsearch by using PF00190 as the query and subsequent domain verification. Among these genes, 50 genes were located in the A_*n*_ subgenome, while the remaining 46 genes were found in the C_*n*_ subgenome. The overall distribution of *BnCDP* genes was uneven across chromosomes (Supplementary Figure 1). Chromosome A02 (seven genes), A06 (eight genes), A07 (eight genes), A09 (six genes) and C08 (11 genes) had the most *BnCDP* genes; while A04 and C02 only contained two and one *BnCDP* gene, respectively; and A05 chromosome even had no *BnCDP*. The length of BnCDP proteins ranged from 175 aa (BnaC08g43590D) to 652 aa (BnaC03g48460D), with an average length of 286 aa. The exon number of each *BnCDP* gene ranged from 1 to 7 (only *BnaA06g25170D*), with most of the members (74%) having no more than three exons. The predicted theoretical pI values varied from 4.77 to 9.77 and the MW values were between 19.45 and 78.20 kDa. Moreover, the GRAVY (grand average of hydrophobicity) index values ranged from –1.300 to 0.545. According to the predicted subcellular location, the BnCDP proteins showed a wide subcellular distribution pattern and were mainly located in extracellular (41/96) and plasma membrane (18/96), and the remaining proteins were specifically located in the chloroplast (11), cytoplasmic region (11), nuclear (7), endoplasmic reticulum (5) and mitochondrion (3) (Supplementary Table 1).

### Phylogenetic analysis of *BnCDPs*

**FIGURE 1:**
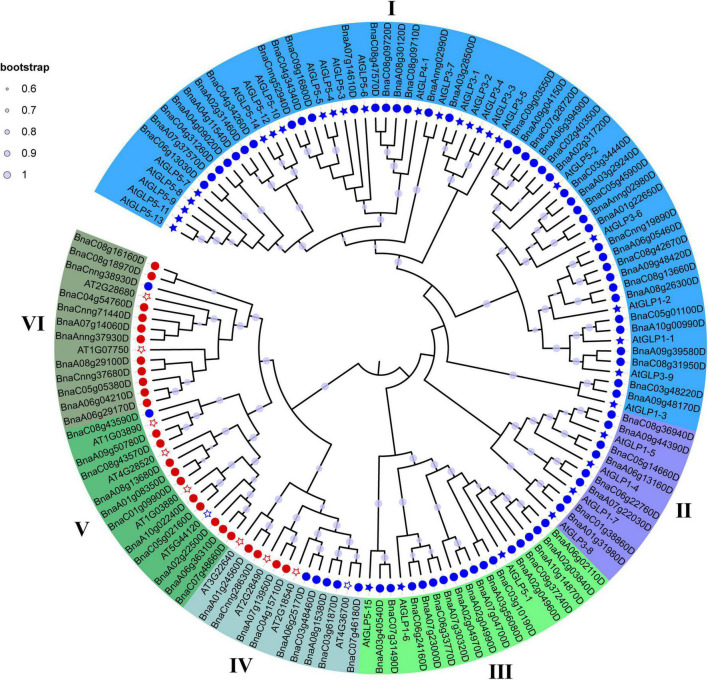
A neighbor-joining (NJ) phylogenetic tree of CDP proteins in *Arabidopsis thaliana* (At) and *Brassica napus* (Bn). All CDP proteins were clustered into six subfamilies, and each subfamily was represented by a different color. Light purple dot on a branch in the figure indicates that the bootstrap support is greater than 60%. Circles refer to BnCDPs, while filled stars refer to AtGLPs identified previously and empty stars refer to AtCDPs identified in this research; Blue and Red refer to CDPs with single or duplicated cupin_1 domain, respectively.

To further characterize and classify the *BnCDP* family members, we constructed a phylogenetic tree using all the 96 BnCDP proteins from *B. napus* and 42 AtCDP proteins from *A. thaliana*. Previous studies have reported 32 *AtGLP* genes in *A. thaliana* (Li L. et al., 2016). In this research, we identified ten new *AtCDP* genes including two monocupin coding genes (*AT4G36700* and *AT5G44120*) and eight bicupin coding genes (*AT2G28490*, *AT3G22640*, *AT1G03880*, *AT1G03890*, *AT4G28520*, *AT1G07750*, *AT2G28680* and *AT2G18540*) in *A. thaliana* (Supplementary Table 2). Finally, these CDP proteins were assigned to I–VI subfamilies based on the topology of the phylogenetic tree, in which the members in subfamily I–III were all monocupins; the members in subfamily V (except for AT5G44120 and BnaC08g43590D) and VI (except for BnaCnng38930D) are almost all bicupins; while subfamily IV was a mixture of monocupins (six) and bicupins (seven; Figure 1 and Supplementary Table 2). The number of *BnCDP* genes varied significantly among the six subfamilies, with subfamily I including approximately half of all the *BnCDP* genes (40), while the subfamily II–VI only comprised 8, 16, 9, 11 and 12 *BnCDP* members, respectively. Except for four genes (*AtGLP1-4*, *AtGLP1-5*, *AtGLP1-7* and *AtGLP3-8*) clustered in subfamily II and three genes (*AtGLP5-1*, *AtGLP1-6* and *AtGLP5-15*) clustered in subfamily III, the remaining previously reported *AtGLP* genes (Li L. et al., 2016) all fell into subfamily I, and all the ten newly identified *AtCDP* members were scattered in subfamily IV (four members), V (four members) and VI (two members). These results indicated that the *CDP* genes were present in the common ancestors of *A. thaliana* and *B. napus*, and some of them might have undergone species-specific expansion and subsequently significant divergence.

**FIGURE 2:**
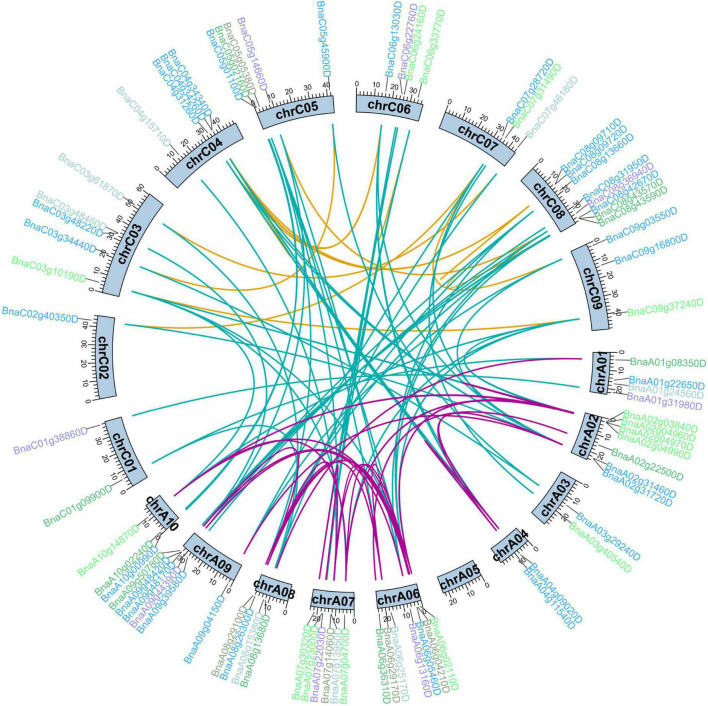
Duplication analysis of *BnCDP* genes in *Brassica napus*. The gene names are marked outward of the corresponding chromosomes. The different colors indicate different subfamilies of the *BnCDP* genes. The duplicated gene pairs are highlighted with connecting lines colored according to the subgenomes, purple indicates that both genes in the gene pair are from the A_*n*_ subgenome, yellow represents that both genes in the gene pair are from the C_*n*_ subgenome, while the blue shows that two genes in the gene pair come from different subgenomes.

Duplication events of the *BnCDP* genes were detected based on BLAST and MCScan X. Briefly, all 96 *BnCDPs* were derived from duplication, among which 72 genes (75.0%) were generated from whole-genome duplication (WGD) or segmental duplication, and 18 genes (18.75%) resulted from dispersed duplication (Supplementary Table 1). Moreover, three tandem and three proximal gene duplication types were detected. There were 191 paralogous gene pairs with high identities (identity > 75%, and alignment length > 75%) in *B. napus*, with 56 gene pairs in the A_*n*_ subgenome, 32 gene pairs in the C_*n*_ subgenome, and the remaining 103 duplication events occurring between the two subgenomes (Figure 2 and Supplementary Table 3). To estimate the selection pressure on *BnCDP* genes in *B. napus*, the ratio of non-synonymous substitution to synonymous substitution (*K*_*a*_/*K*_*s*_) for the 191 paralogous gene pairs was calculated. The results showed that the *K*_*a*_/*K*_*s*_ ratio for all paralogous gene pairs varied from 0 to 0.52 and lower than 1, suggesting that the *BnCDP* genes have undergone purification selection during evolution (Supplementary Table 3).

### Gene structure and conserved motif analysis of the *BnCDP* family in *Brassica napus*

**FIGURE 3:**
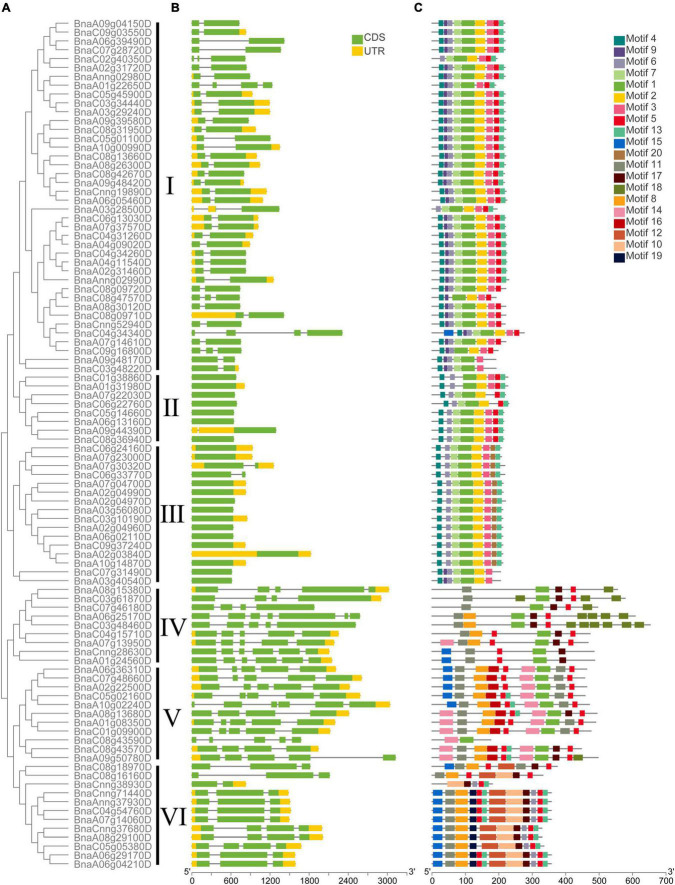
The phylogenetic relationship, exon-intron architecture, and conserved motifs of 96 BnCDP in *Brassica napus*. **(A)** The phylogenetic relationships of BnCDP proteins based on the NJ method. **(B)** Gene structures of *BnCDP* genes. Yellow boxes represent the untranslated region (UTR), green boxes represent exons and the gray lines represent introns. **(C)** The conserved motif composition of BnCDP proteins. Scale bars represent gene length (bp) and protein sequence length (aa).

The exon-intron structure of all the *BnCDP* genes in six subfamilies was displayed based on their phylogenetic relationships (Figures 3A,B). As shown in Figure 3B, 36 members contained both 5′ and 3′ UTRs; 33 members exhibited no UTR; while the remaining 27 members possessed either a 5′ or 3′ UTR. Furthermore, the gene structure seemed to vary remarkably among different subfamilies, but were relatively conserved within the subfamily. For example, most *BnCDPs* in subfamily I had two exons, and all members in subfamily II and nearly all members in subfamily III were intronless. Most of members in subfamily VI had three exons, while members in subfamily IV had the maximum number of exons and introns.

We also analyzed the distribution of conserved motifs in the *BnCDP* family. In total, 20 distinct conserved motifs were identified (Figure 3C and Supplementary Table 4). In subfamily I–III, the motifs were well conserved. Most genes in these subfamilies simultaneously contained motif 1, 2, 3, 4, 7. However, motif 9 was specifically identified in members in subfamily I except for two genes in subfamily III (*BnaC07g31490D* and *BnaA03g40540D*). Members in subfamily III specifically contained motif 20 but were lack of motif 5 prevalently present in other five subfamilies. Motif 10, 12 and 19 were only found in subfamily VI, and motif 11 was present in nearly all members in subfamily IV–VI. The specificity of motifs was conformed to the observed evolutionary characteristics of *BnCDP* genes, implying that the specifically conserved motifs in different *BnCDP* subfamilies are associated with their functions.

### Analysis of *cis*-regulatory elements in the promoter region of *BnCDPs*

**FIGURE 4:**
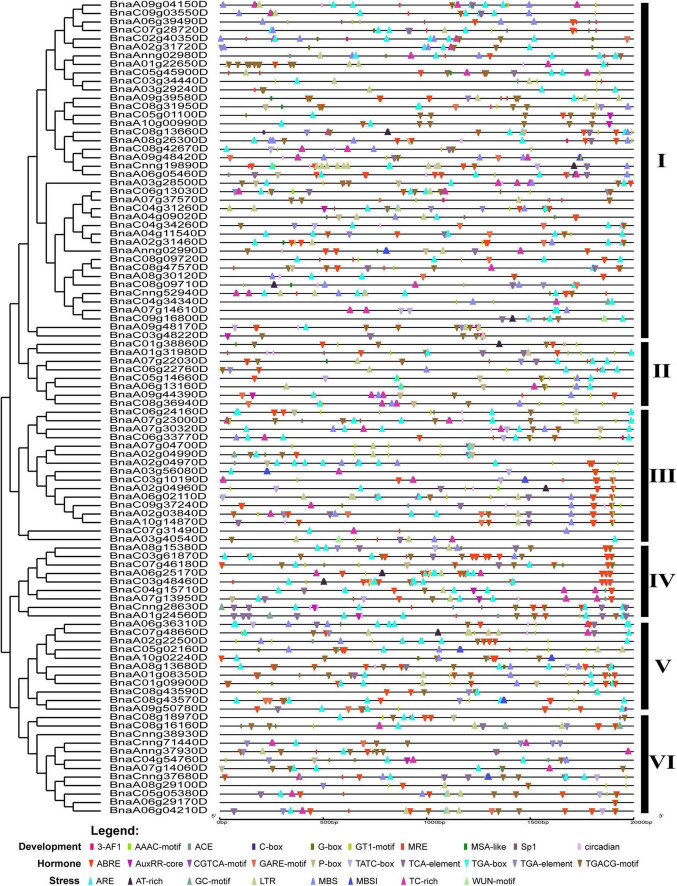
*Cis*-acting regulatory elements identified in promoters of *BnCDP* genes in *Brassica napus*. Boxes indicate development-related elements, down-wedges indicate hormone-related elements, and up-wedges indicate stress-related elements. Different colors indicate different elements.

The regulatory elements in the promoter region are often relatively conserved in sequence and function throughout evolution, particularly in tolerance to biotic and abiotic stresses (Oudelaar and Higgs, 2021). To explore the potential roles of *BnCDP* genes, we analyzed the *cis*-regulatory elements in the 2-kb promoter regions of *BnCDPs* based on the PlantCARE database. As a result, the *cis*-acting regulatory elements associated with development, hormone and stress were enriched in these promoters (Figure 4, Supplementary Figure 2, and Supplementary Tables 5, 6). These *cis*-regulatory elements included development related elements such as the circadian element (involved in circadian control), the GT1-motif, Sp1, ACE, G-box and GT1 motif (involved in light responsiveness); hormone responsive elements such as ABRE (involved in the abscisic acid response), AuxRR-core and TGA-element (involved in auxin response), GARE-motif and TATC-box (involved in gibberellin response), CGTCA-motif and TGACG-motif (involved in MeJA response) and the TCA-element (involved in salicylic acid response); stress-responsive elements such as ARE elements (essential for anaerobic induction), TC-rich repeats (involved in defense and stress responsiveness), AT-rich sequence (element for maximal elicitor-mediated activation), LTR (involved in low-temperature responsiveness), MBS (MYB binding site involved in drought-inducibility), MBSI (involved in flavonoid biosynthesis) and WUN-motif (involved in wound response). There were many MeJA-related *cis*-acting regulatory elements in the promoters of *BnCDP* genes: the CGTCA-motif was identified in 71 of the 96 *BnCDP* gene promoters (2.13 on average for each promoter), and the ABRE elements were present in 75 *BnCDP* promoters (with 3.39 on average for each promoter). Besides, 65 TC-rich repeats associated with defense and stress response were unevenly scattered in 45 *BnCDP* gene promoters, which were the most enriched in cluster III (10/16) and VI (8/12), but were rare in cluster V (2/11), while 83 low-temperature response (LTR) elements showed relatively unbiased distribution in subfamily I–VI. Moreover, ARE (84/96, 87.50%) and MBS (49/96, 51.04%) elements were also common in the promoters of *BnCDP* genes. These results implied that members of the *BnCDP* family are potentially involved in biotic and abiotic stress response during plant growth and development.

The spatiotemporal expression patterns of the genes playing essential roles in plant development and stress responses tend to be regulated by their corresponding transcription factors (TFs) (Jin et al., 2014). Hence, we analyzed the transcription factor binding sites (TFBSs) in the promoter regions of all 96 *BnCDP* genes, and found that 64 of the promoters contain TFBSs, which correspond to 21 TF families (Supplementary Table 7). These TFs included Dof, B3, AP2, MIKC_MADS, MYB, MYB_related, GATA, ERF, C2H2, LBD, GRAS, Nin-like, BBR-BPC, SRS, NAC, E2F/DP, bZIP, ARF, bHLH, SBP, and Trihelix. Among the 64 promoters, 22 and 42 potentially bind to single and multiple TFs, respectively (Supplementary Table 7).

### Expression patterns of *BnCDP* genes in multiple tissues of the whole growth period

**FIGURE 5:**
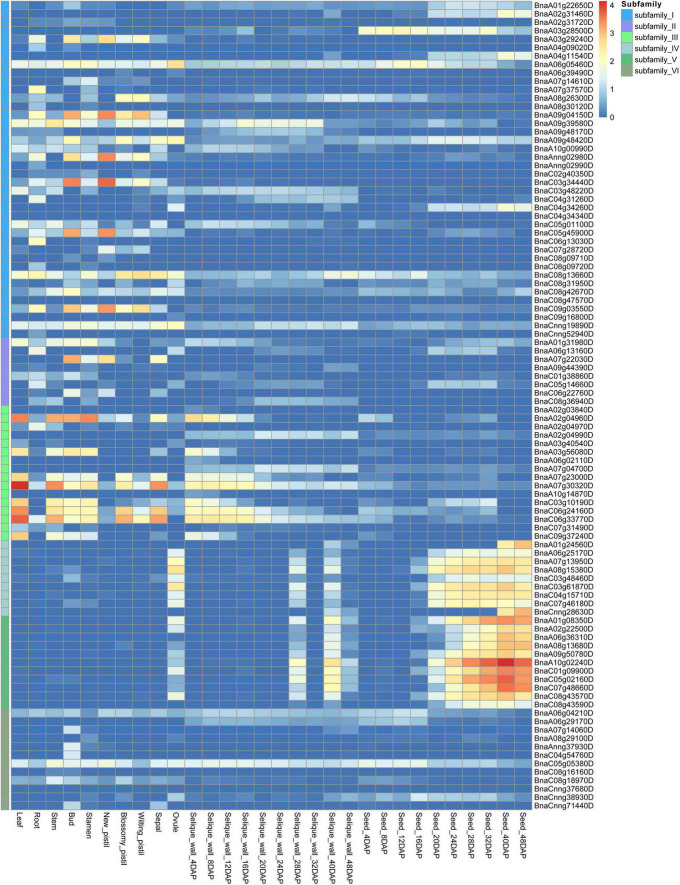
Expression patterns of 96 *BnCDP* genes in 30 tissues of *Brassica napus* ZS11 variety. The expression data were processed with the log_10_ normalization of fragments per kilobase million (FPKM). The color scale represents relative expression levels from low (blue color) to high (red color).

To comprehensively explore the potential function of *BnCDP* genes, we investigated the expression patterns of all 96 *BnCDP* members in 30 tissues/stages, including the leaf, root, stem, bud, stamen, new pistil, blossomy pistil, wilted pistil, sepal, ovule and ten time-course seeds and silique walls [from 4 to 48 days after pollination (DAP)] based on our previously published transcriptome data (Figure 5 and Supplementary Table 8; He et al., 2022). Overall, 89 genes were expressed (FPKM > 1) in at least one tissue or stage, with 48 genes showing high expression (FPKM > 50), 24 genes exhibiting intermediate expression (FPKM > 10) and three genes being lowly expressed (FPKM < 10). Generally, most of the *BnCDP* genes showed highly tissue-specific expression patterns, and only three genes (*BnaC08g13660D*, *BnaCnng19890D* and *BnaC05g05380D*) were expressed in all the 30 tissues or stages (Figure 5). Members in subfamily IV and subfamily V displayed nearly identical expression patterns, which were highly expressed in the ovule and silique wall (28 and 40 DAP), and had extremely high expression in the seed at the intermediate to late stages, implying their essential roles in the successful reproduction of *B. napus*. The genes in subfamily I and subfamily II showed great differences in expression and some of them were highly expressed in the root, bud and pistil. Overall, members in subfamily III showed high expression levels, such as in the leaf, stem and early stages of silique wall development, particularly *BnaA02g04960D*, *BnaA07g23000D*, *BnaA07g30320D*, *BnaC06g24160D* and *BnaC06g33770D*; while the members in subfamily VI exhibited relatively low expression levels. These differences in expression pattern among subfamilies implied that the *BnCDP* genes may be involved in tissue- or stage-specific developmental processes.

### Expression patterns of *BnCDP* genes under biotic and abiotic stresses

**FIGURE 6:**
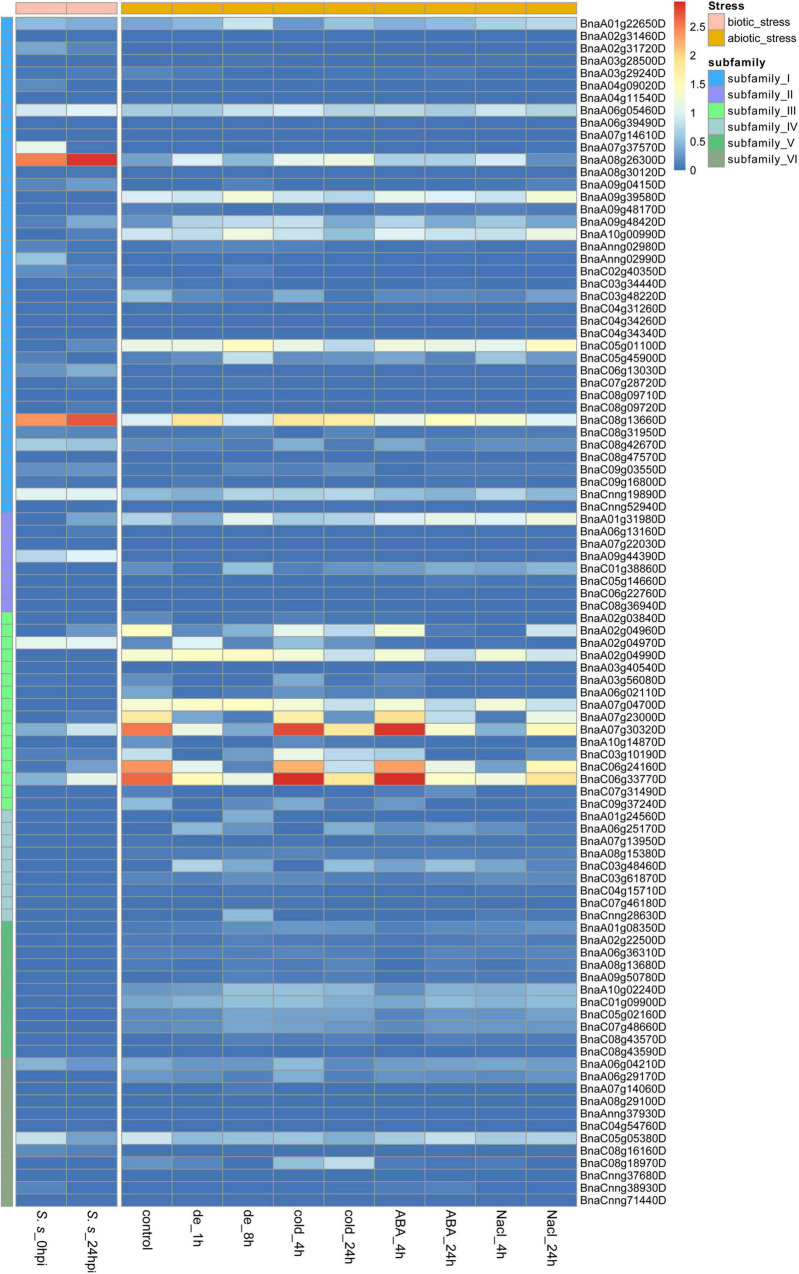
Expression profiles of 96 *BnCDP* genes under biotic (left) and abiotic (right) stress conditions. The left panel shows the expression level of *BnCDP* genes in ZY821 at 0 and 24 h after *Sclerotinia sclerotiorum* inoculation; The left panel shows the expression level of *BnCDP* genes under different abiotic stress conditions (dehydration, cold, ABA and salinity). The expression data were processed with the log_10_ normalization of fragments per kilobase million (FPKM). The color scale represents relative expression levels from low (blue color) to high (red color).

As sessile organisms, the growth and development of plants are constantly challenged by various stresses. *S. sclerotiorum* is an ascomycete plant pathogen causing Sclerotinia stem rot in *B. napus* and severely affecting its seed yield and quality. To analyze the role of *BnCDPs* in the response to biotic stress, we investigated the expression profiles of 96 *BnCDPs* in the leaves of tolerant variety ZY821 of *B. napus* at 0 and 24 h after inoculation with *S. sclerotiorum* (Girard et al., 2017). Interestingly, just a part of *BnCDP* members were responsive to *S. sclerotiorum* inoculation, as many of them were inactive in the leaves of ZY821, such as all members in subfamily IV and V, which had no transcript accumulation at all (Figure 6). Among the 18 expressed *BnCDP* genes (FPKM > 1), 13 exhibited remarkable expression changes at 24 h post-inoculation (hpi), with eight genes (*BnaA08g26300D*, *BnaA09g48420D*, *BnaC06g13030D*, *BnaC08g13660D* from subfamily I; *BnaA09g44390D* from subfamily II; and *BnaA07g30320D*, *BnaC06g24160D*, *BnaC06g33770D* from subfamily III) being up-regulated and five genes (*BnaA02g31720D*, *BnaA07g37570D*, *BnaAnng02990D* from subfamily I; and *BnaA06g04210D*, *BnaC05g05380D* from subfamily VI) being down-regulated. Particularly, *BnaA08g26300D* and *BnaC08g13660D* in subfamily I exhibited high expression (FPKM > 500) and heavily induced by *S. sclerotiorum* inoculation in ZY821 (Figure 6 and Supplementary Table 9).

To identify the potential functions of *BnCDP* genes in response to different abiotic stresses, we analyzed the RNA-seq data from samples under dehydration, cold, ABA and salinity treatment, respectively (Zhang et al., 2019). By taking two-fold change as the threshold value, we identified and compared the differentially expressed genes under each of the above-mentioned stresses (Figure 6 and Supplementary Table 10). Under dehydration treatment, 25 out of the 29 expressed *BnCDP* genes (FPKM > 1) were significantly responsive at 1 h and/or 8 h of treatment, and most of upregulated genes (9/12) were from subfamily I, while the downregulated genes (8/12) were mainly from subfamily III. Besides, one gene (*BnaA01g31980D*) in subfamily II displayed an opposite pattern, which was downregulated at 1 h of treatment, but subsequently upregulated at 8 h. Under cold treatment, 29 *BnCDPs* showed differential expression, and more than two-thirds (21/29) of them belong to subfamily I and III. A total of 20 *BnCDP* genes were significantly responsive to ABA treatment and almost all down-regulated genes (8/9) belonged to subfamily III. Twenty-four *BnCDP* genes showed significant fold changes in response to NaCl treatment, and 90% of the responsive genes (9/10) in subfamily I were upregulated except for *BnaC03g48220D*, while all ten responsive members in subfamily III were downregulated. The transcript levels of genes from subfamily VI almost showed no fluctuation during ABA and NaCl treatment. Overall, the *BnCDP* family showed highly similar expression patterns in response to the above four abiotic stresses, and mainly the members in subfamily I and subfamily III were responsive to the treatments: The former tended to be induced while the latter was usually down-regulated in response to these stresses. Besides, we found that 13 *BnCDPs* were significantly responsive to all the four abiotic stresses, among which six members (*BnaA07g23000D*, *BnaA07g30320D*, *BnaA08g26300D*, *BnaC06g24160D*, *BnaC06g33770D* and *BnaC08g13660D*) were involved in response to *S. sclerotiorum* inoculation (Supplementary Figure 3). These genes are common stress-responsive genes shared by multiple biotic and abiotic stresses and may be used for the breeding of varieties with multiple stress resistance in *B. napus*.

### Genome wide association study on *Sclerotinia sclerotiorum* resistance and functional candidate gene *BnGLPs* analysis

**FIGURE 7:**
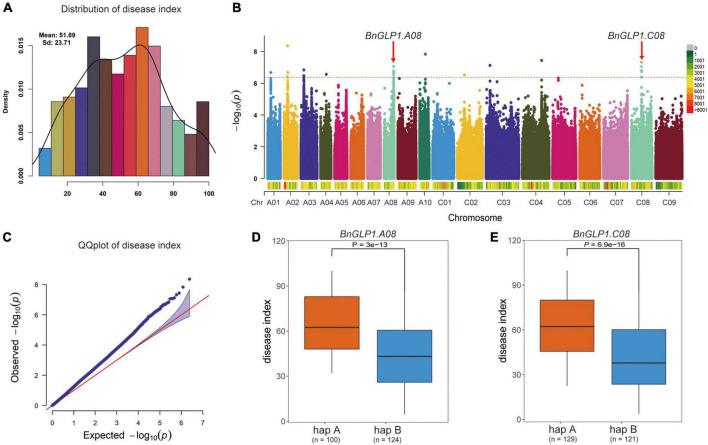
Both *BnGLP1.A08* and *BnGLP1.C08* are associated with *Sclerotinia sclerotiorum* resistance in *Brassica napus*. **(A)** Frequency distribution of disease index of 274 *B. napus* accessions. **(B)** Manhattan plot of the disease index from association analyses by FarmCPU model. Each point represents a SNP, and the SNP that exceeds the threshold (red dotted line) –log_10_ (1/*n*) = 6.377 is significant. **(C)** QQ plot for the disease index from association analyses. **(D,E)** Boxplots for disease index based on the two haplotypes of *BnaGLP1.A08* **(D)** and *BnaGLP1.C08* **(E)**. The phenotypic differences between groups were tested using a two-tailed *t*-test (*P* < 0.01).

To further examine the potential effects of *BnCDP* genes in *S. sclerotiorum* resistance, we used more than 2.38 million SNPs with MAF > 0.05 across 274 worldwide collected accessions to perform a GWAS based on disease index using the FarmCPU model. As shown in Figure 7A, the frequency distribution of disease index approximates to normal distribution, indicating that this population was suitable for association analysis. Then, the SNP GWAS was conducted and the significant SNPs associated with *S. sclerotiorum* resistance were displayed on Manhattan plot (Figure 7B) and QQ plot (Figure 7C). A total of 24 significant SNP loci (*P* < 4.199 × 10^–7^; Bonferroni-adjusted significance threshold 1/*n*, *n* = 2,381,566; Figure 7B) associated with *S. sclerotiorum* resistance were detected, which constituted 10 QTL distributed on ten chromosomes including A01, A02, A03, A04, A08, A10, C03, C03, C04 and C08 of *B. napus*. Among them, four QTL containing five *BnGLPs* (*BnaA04g11540D*, *BnaA08g26300D*, *BnaC04g34260D*, *BnaC04g34340D* and *BnaC08g13660D*) were identified. Furthermore, *BnaA08g26300D* and *BnaC08g13660D* are WGD genes (ortholog of *AT1G09560*/*AtGLP1-2*), while *BnaA04g11540D* and *BnaC04g34260D* are WGD genes (ortholog of *AT5G39130*/*AtGLP5-10*). Considering that *BnaA08g26300D* and *BnaC08g13660D* (named as *BnGLP1.A08* and *BnGLP1.C08*, respectively) gene pairs are both common responsive genes for biotic and abiotic (dehydration, cold, ABA and salinity treatment) stresses, we took the two genes as candidates for further haplotype analysis. Our results revealed that each of *BnGLP1.A08* and *BnGLP1.C08* had two major haplotypes associated with *S. sclerotiorum* resistance (Figures 7D,E).

**FIGURE 8:**
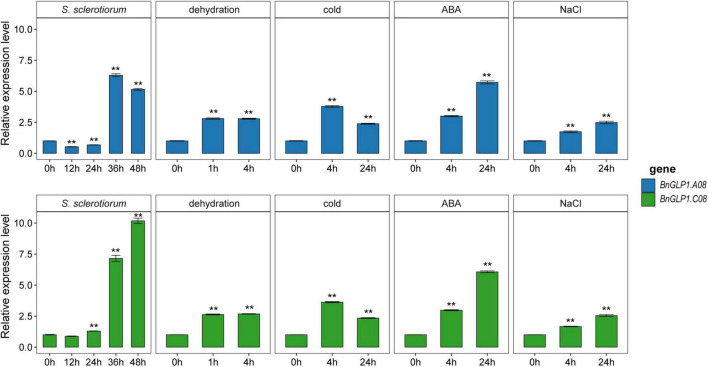
The expression validation of candidate *BnGLP1.A08* and *BnGLP.C08* genes in response to *Sclerotinia sclerotiorum* infection and four abiotic stress treatments (dehydration, cold, ABA and salinity) by qRT-PCR. The time points under the x-axis represent hours (h) after corresponding biotic and abiotic treatments. The error bars show the standard error of three replicates. Student’s *t*-test was used for statistical analysis, ** indicates significant differences at *P* < 0.01, all compared to the treatment at 0 h.

To confirm that *BnGLP1.A08* and *BnGLP1.C08* are involved in adaptive stress response, we determined their expression levels of *B. napus* seedlings under *S. sclerotiorum* infection and different abiotic stress treatments (dehydration, cold, ABA and salinity) by quantitative real-time PCR (qRT-PCR). The leaves at four time points (12 h, 24 h, 36 h and 48 h) after *S. sclerotiorum* inoculation were collected to explore the dynamic gene expression changes (Figure 8). The results demonstrated that the expression pattern of *BnGLP1.A08* and *BnGLP1.C08* after *S. sclerotiorum* inoculation was very similar, which stayed relatively consistent during the first 24 h and was then upregulated to a peak (Figure 8). For abiotic stress responses, the expression level of both *BnGLP1.A08* and *BnGLP1.C08* genes showed significantly increased expression under dehydration, cold, ABA or salinity treatment, and displayed more sensitive to ABA treatment, leading to more accumulation in transcripts (Figure 8). Taking together, the qRT-PCR analysis verified the reliability of the RNA-seq data and revealed that both *BnGLP1.A08* and *BnGLP1.C08* gene pairs are common responsive genes shared by multiple stresses.

## Discussion

Environmental stresses such as pathogen infection or drought, salinity, heat and cold cause devastating impacts on plant growth and extensive losses in the crop yield (Suzuki et al., 2014). Sclerotinia stem rot caused by *S. sclerotiorum* is a devastating disease leading to significant yield and economic losses in many crop and vegetable plants, particularly *Brassica* crops (Liu et al., 2021). Moreover, drought and salt stresses are important limiting factors affecting about 26% and 20% of the agricultural land, respectively (Raza et al., 2021). Therefore, it is urgent to identify more effective loci with durable disease resistance or diverse abiotic stress tolerance in plants, especially in important agricultural crops, to develop biotic and abiotic stress-tolerant genotypes. Single cupin_1 domain GLPs play essential roles in regulating plant development and biotic/abiotic stress resistance (Dunwell et al., 2004). However, the prevalence and functional diversity of the *CDP* gene family in *B. napus* have not been thoroughly investigated. In this study, we performed a comprehensive analysis of the *BnCDP* family in *B. napus*. The features of *BnCDP* genes, including their chromosomal distribution, phylogenetic classification, gene structures, conserved motifs, *cis*-regulatory elements, expression profiles, and responses to various stresses were explored. The results will provide insights into this gene family and offer valid information for predicting their potential functions in plant growth and stress response.

To date, the *GLP* genes (encoding single cupin_1 domain protein) have been genome-wide identified in numerous plant species, such as *A. thaliana* (Li L. et al., 2016), rice (Li L. et al., 2016), soybean (Lu et al., 2010), *Vitis vinifera* (Ilyas et al., 2022), cucumber (Liao et al., 2021), potato (Zaynab et al., 2021), peanut (Wang et al., 2013) and *Physcomitrella patens* (Nakata et al., 2004). However, the genes encoding duplicated cupin_1 domain protein were till rarely characterized. In this study, 96 *BnCDPs* in *B. napus* and 42 *AtCDPs* (including 32 previously identified *AtGLP*s) were systematically identified. Gene duplication might account for the differences in the number of *CDP* family members between *B. napus* and *A. thaliana*. In our results, 75.0% of *BnCDPs* (72 *BnCDP* genes) were originated from WGD or segmental duplication, which is consistent with the conclusion that WGD and segmental duplication are the main contributors to the expansion of gene families in other researches (Ma et al., 2017; Wu et al., 2018; Xie et al., 2022). Since *B. napus* was formed by the interspecific hybridization between *B. rapa* and *B. oleracea* about 7,500 years ago, both of which had undergone a genome triplication event after divergence from *A. thaliana* lineage, six homologs for each *A. thaliana* gene are expected to be present in *B. napus* (Allender and King, 2010). However, the number of identified *BnCDPs* was much smaller than expected (less than threefold of *AtCDPs*), which may be ascribed to the occurrence of gene loss during the diploidization process (Albalat and Canestro, 2016). Despite the uneven distribution of *BnCDP* genes at the chromosome level, the total number of genes was roughly similar in the A_*n*_ (50 members) and C_*n*_ subgenome (46 members) (χ^2^ = 0.167 < 3.84). Based on the *K*_*a*_/*K*_*s*_ ratio of paralogous gene pairs (Supplementary Table 3), it can be speculated that purification selection plays a significant role in the evolution of *BnCDP* genes in *B. napus.*

A phylogenetic analysis of the CDP members from *B. napus* and *A. thaliana* revealed that these CDPs could be divided into six subfamilies (I–VI). All members in subfamily I–III are GLPs, while those in subfamily IV–VI tend to be bicupins, and their monocupin members showed closer evolutionary relationship with bicupins than with other GLPs (Figure 1). Dunwell et al. predicted that bicupins probably evolved from the duplication and then fusion of a single domain ancestor (Dunwell et al., 2004). Our phylogenetic analysis indicated that the fused duplicated domain protein (bicupin) might also lose a domain during the subsequent evolution to produce a new monocupin with higher similarities in sequence and gene structure to its bicupin ancestor. Furthermore, *BnCDPs* within the same subfamily have high similarities in gene structure and motif distribution (Figure 3), implying that the members in the same subfamily have similar functions.

Spatio-temporal expression pattern can reflect the potential function of a gene to a certain extent. In the present study, we analyzed the expression patterns of all 96 *BnCDP* members in 30 tissues/stages (He et al., 2022). As shown in Figure 5, most of the *BnCDP* genes showed preferential expression in specific tissues/stages, which is consistent with the previous findings in cucumber, rice and *A. thaliana* (Li L. et al., 2016; Liao et al., 2021). For example, almost all members in subfamily IV and subfamily V were predominantly expressed in seeds at the intermediate to the late stage; *BnaA09g04150D*, *BnaAnng02980D* and *BnaC03g34440D* from subfamily I were predominantly expressed in the bud and pistil; *BnaA07g22030D* from subfamily II was highly expressed in the pistil; *BnaA07g30320D* and *BnaC06g33770D* from subfamily III showed high accumulation of transcripts in the leaf. These results indicate that they play essential roles in these tissues/stages. Besides, three genes (*BnaC08g13660D*, *BnaCnng19890D*, *BnaC05g05380D*) were expressed in all the 30 tissues/stages (Figure 5), suggesting their possible essential roles in the entire growth and development stages.

Plant *GLP* gene*s* also play vital roles in regulating biotic and abiotic stress responses. Numerous studies have demonstrated that the *GLPs* are widely involved in resistance to diverse pathogens such as *S. sclerotiorum* (Rietz et al., 2012; Zhang et al., 2018), *Rhizoctonia solani* (Beracochea et al., 2015), *Blumeria graminis* (Yuan et al., 2021), *Magnaporthe oryzae* (Liu et al., 2016), *Aspergillus flavus* (Wang et al., 2013), and response to UV-B radiation (He et al., 2021), heat (Gangadhar et al., 2021; Zaynab et al., 2021), drought (Wang et al., 2013; Anum et al., 2022), heavy metal (Cheng et al., 2018) and wound (Wang et al., 2013). To further explore the possible function of *BnCDPs* in stress resistance, the analysis of *cis*-acting regulatory elements in the promoter regions was conducted in this study. The results revealed the enrichment of elements associated with development, hormone and stress (Figure 4, Supplementary Figure 2 and Supplementary Table 5), implying that the members of the *BnCDP* family are potentially involved in biotic and abiotic stress responses during plant growth and development. Consistently, many members in the *BnCDP* family were significantly responsive to one or more stresses according to the transcriptome data under one biotic stress (*S. sclerotiorum* infection) and four abiotic stresses (dehydration, cold, ABA and salinity). Furthermore, six *BnCDP* genes were commonly regulated by *S. sclerotiorum* infection and all the four abiotic stresses, which belong to multiple biotic and abiotic stress-responsive genes. Similar results were previously obtained that several members in both barley *HvGER* family (Zimmermann et al., 2006) and Peanut *AhGLP* family (Wang et al., 2013) appeared to participate in multiple biotic and abiotic stress responses. Notably, the *bicupins* from subfamily IV–VI are also responsive to multiple stresses, such as *BnaCnng28630D* in subfamily IV responsive to dehydration treatment, *BnaA10g02240D* in subfamily V responsive to dehydration, cold and ABA treatments, *BnaC05g05380D* in subfamily VI responsive to dehydration, cold treatments and *S. sclerotiorum* infection. These results suggested that some other *BnCDP* genes apart from *BnGLPs* may also have important functions in environmental adaption, and some of them were promising broad-spectrum stress resistance candidates with tremendous potential in improving crop resistance to different stresses. To further examine the potential roles of *BnCDP* genes in *S. sclerotiorum* resistance, we performed a GWAS analysis. The results showed that five *BnGLPs* are located in the significant associated regions, including a duplicate gene pair *BnGLP1.A08* and *BnGLP1.C08*, whose response patterns to different biotic and abiotic stresses were validated by qPCR experiment. The results support the conclusion that the *BnCDP* family members are widely involved in environmental adaption of *B. napus*. This study provides a useful resource for future research on the biological function and evolutionary history of the *BnCDP* gene family.

## Conclusion

In this study, the cupin_1 domain protein (CDP) gene family in *B. napus* was genome widely characterized and systematically investigated. In total, 96 *BnCDP* genes were identified and clustered into six distinct subfamilies (I–VI) based on their evolutionary relationships. Genes from the same subfamily have similar gene structure and motif distribution, which are more conserved in subfamily I–III (BnGLP) than in subfamily IV–VI. To better understand their potential functional roles, we analyzed the *cis*-regulatory elements and TFBSs in the promoters of *BnCDP*s, as well as their expression patterns in diverse tissues/stages and under various biotic and abiotic stresses. The results demonstrated that the *BnCDP* family members play important roles in plant development and stress tolerance, particularly the six genes commonly regulated by *S. sclerotiorum* infection and four abiotic stresses, which may serve as promising broad-spectrum stress tolerance candidates. GWAS on *S. sclerotiorum* resistance revealed that two (*BnGLP1.A08* and *BnGLP.C08*) of the six common stress response candidate genes were located in significant associated regions, and their expression patterns under different biotic and abiotic stress treatments were further validated by qPCR analysis. In summary, this study provides detailed information about *BnCDP*s in *B. napus*, and will facilitate the functional studies and genetic improvement to deal with different stresses.

## Materials and methods

### Identification of *BnCDP* gene family in *Brassica napus*

The genome (v4.1) and annotation (v5) information of the *B. napus* cultivar “*Darmor-bzh*” (Chalhoub et al., 2014) was obtained from the Brassicaceae Database (BRAD^2^). To identify BnCDPs in *B. napus*, PF00190 from the Pfam database^3^ (Mistry et al., 2021) was used as a query to search in the entire protein database of *B. napus* using HMMER 3.3.2^4^ (Mistry et al., 2013; the e-value was set to 1 × 10^–5^). Then, all putative BnCDPs identified were subjected to the NCBI Conserved Domain Database^5^ (Lu et al., 2020) and the SMART database^6^ (Letunic et al., 2021) to verify the presence of cupin_1 domain. Moreover, the peptide length, molecular weight (MW), isoelectric point (pI), instability index, gravy of each BnCDP proteins were calculated using ProtParam^7^ (Wilkins et al., 1999), an online software of SWISS-PROT. Subcellular location prediction for these BnCDP proteins was conducted using CELLO v2.5^8^ (Yu et al., 2006). The physical locations on the chromosomes and the exon number of the *BnCDP* genes were obtained from the GFF3 annotation file of the *B. napus* genome using a custom shell script.

### Phylogenetic analysis

To gain insights into the evolutionary relationships of *BnCDP* genes in *B. napus*, the amino acid sequences of all 96 BnCDPs identified in this study, combined with 32 previously identified *A. thaliana* GLPs (Li L. et al., 2016) and 10 newly identified cupins, were subjected to multiple sequence alignment using ClustalW2 program (Larkin et al., 2007) with default parameters. Then, MEGA 11 (Tamura et al., 2021) software was used to generate the phylogenetic tree using the neighbor-joining (NJ) method with 1,000 bootstrap replicates. The final phylogenetic tree was visualized using iTOL v6.^9^ The *BnCDP* genes were further categorized into different subfamilies based on the topology of the phylogenetic tree.

### Chromosomal distribution and gene duplication analysis of *BnCDP* genes

To identify gene duplication events, BLASTP with the e-value of 1e–10 was used to align the sequence, and MCScanX (Wang et al., 2012) was used to detect the duplication patterns including segmental and tandem duplication. Chromosomal locations and duplication events were visualized using the TBtools software (Chen et al., 2020). To determine the evolutionary pressure on duplicated genes, the ratio of non-synonymous substitution to synonymous substitution (*K*_*a*_/*K*_*s*_) of duplicate gene pairs was calculated using TBtools (Chen et al., 2020).

### Gene structure, conserved motif, and *cis*-regulatory element analysis

The conserved motif analysis of BnCDPs was conducted using the online motif finding tool, MEME (Multiple Expectation Maximization for Motif Elicitation, v5.4.1^10^; Bailey et al., 2015) with 20 motif numbers, and the remaining parameters were set to default values. The identified motifs were annotated by using the Interpro database.^11^ The TBtools (Chen et al., 2020) software was used to display the gene structures and conserved motifs in BnCDP proteins. To identify the *cis*-regulatory elements of *BnCDP* genes, the promoters (2-kb upstream sequences from initiation codon) of *BnCDPs* were extracted and predicted by PlantCARE^12^ (Lescot et al., 2002). The location and type of each selected *cis*-regulatory element were displayed by Gene Structure Display Server (GSDS 2.0; Hu et al., 2015). Besides, the transcription factor binding sites (TFBSs) in the promoter region of *BnCDP* genes were predicted using PlantRegMap/PlantTFDB v5.0^13^ and the threshold *p*-value was set to 1e-7.

### Expression analysis of *BnCDP* genes in *Brassica napus*

To explore the spatial-temporal expression patterns of *BnCDPs*, transcriptome data from 30 tissues/stages which include leaf, root, stem, bud, stamen, new pistil, blossomy pistil, wilted pistil, sepal, ovule and ten time-course seeds and silique walls (4, 8, 12, 16, 20, 24, 28, 32, 40, 48 days after pollination) of ZS11 were used in this study (He et al., 2022). Furthermore, in order to detect the expression patterns of *BnCDP* genes under biotic and abiotic stresses, RNA-seq data from tolerant *B. napus* cultivar ZY821 under the induction of *S. sclerotiorum* fungi and different abiotic stress conditions (dehydration, cold, ABA and salt) of *B. napus* cultivar ZS11 were also used in this study (Girard et al., 2017; Zhang et al., 2019). Then, the RNA-seq reads from each sample were aligned to the reference genome of *Darmor-bzh* (v4.1) using Hisat2 (Kim et al., 2015). Subsequently, the expression levels of *BnCDP* genes were calculated with Stringtie (Pertea et al., 2015) and displayed by Pheatmap in R.

### Ribonucleic acid isolation and quantitative real-time PCR analysis of *BnCDP* genes

For *S. sclerotiorum* inoculation, the seedlings of ZY821 were kept growing in greenhouse at 22°C with a 16-h light and 8-h dark photoperiod for 6 weeks. *S. sclerotiorum* isolate obtained from Wuhan field was cultured on potato dextrose agar (PDA) medium and sub-cultured twice before inoculation at 22°C in darkness. Mycelial agar plug (7 mm in diameter) punched from the margin of a 2-day-old culture of *S. sclerotiorum* grown on PDA was carefully upended onto the adaxial surface of the latest or penultimate fully extended leaves with similar size. The inoculated plants were placed in a humidification chamber with high relative humidity (>85%) and samples were taken every 12 h. For abiotic stress treatments, the seeds of ZS11 were sterilized in 75% ethanol for 1 min, in 3.1% NaOCl for 10 min, and then rinsed six times with sterile water. Next, the seeds were sowed on Murashige and Skoog (MS) medium (MS, 1% sucrose, 0.7% agar, pH 5.8) in plates. The plates were placed vertically in the growth chamber with the temperature of 22°C and photoperiod of 16 h/8 h day/night. Two-week-old uniform plants/seedlings were removed from MS medium and subjected to dehydration, low temperature (4°C), ABA (25 uM), and salt (200 mM) stress treatment according to previously described methods (Zhang et al., 2019). Whole seedlings were collected at 1 h and 4 h after dehydration while 4 h and 24 h of low temperature, ABA, and salt treatment. All the samples mentioned above were flash frozen in liquid nitrogen and store at –80°C. Total RNA was extracted using the TRIZOL reagent (Invitrogen) according to the manufacturer’s protocol and subjected to reverse transcription with the PrimeScript RT Reagent Kit with genomic DNA Eraser (Takara). Quantitative Real-time PCR (qRT-PCR) was performed by using SYBR Green Real-time PCR Master Mix (Bio-Rad) in 20 ml reaction mixture and run on CFX96 Real-time PCR system (Bio-Rad). β-actin gene was used as internal control. All the results were obtained with three biological replications, and each with three technical replications. The results were analyzed using the 2^–ΔΔ*CT*^ method as described previously (Livak and Schmittgen, 2001). The list of all the primers used in this study is included in Supplementary Table 11.

### Genome wide association study on *Sclerotinia sclerotiorum* resistance

We selected 274 *B. napus* core germplasm accessions from all over the world to form a natural population. The genotypic data were obtained by 7 × re-sequencing and referring to the genome of ‘Darmor-*bzh*’ (Ding et al., 2020). SNPs were tested using the Broad Institute’s opensource Genome Analysis Toolkit.^14^ Then, the sites with SNP deletion of more than 0.9 or with minor allele frequency (MAF) less than 0.05 were filtered using VCFtools (Danecek et al., 2011), and finally 2,381,566 SNPs were obtained for GWAS. The phenotypic data were collected by investigating the disease index of mature rapeseeds grown at the Yangluo test base (Wuhan, China) from 2015 to 2018 (Ding et al., 2020). GWAS was performed for *S. sclerotiorum* resistance using Fixed and random model Circulating Probability Unification (FarmCPU) model (Yin et al., 2021), and the significance threshold was set to *p* < 4.199 × 10^–7^.

## Data availability statement

The original contributions presented in this study are included in the article/Supplementary material, further inquiries can be directed to the corresponding author.

## Author contributions

YH, YL, and YZ designed the research. SL supervised the research. YH, YL, RZ, and JL performed the experiments. YH, YL, ZB, and MX analyzed the data. CT collected the data. XC and YYL provided the plant materials. YH and YL wrote the manuscript. YZ revised the manuscript. All authors have read and approved the current version of the manuscript.

## Footnotes

1
https://www.fao.org/faostat/

2
http://brassicadb.cn/

3
http://pfam.xfam.org

4
http://www.hmmer.org/

5
https://www.ncbi.nlm.nih.gov/

6
http://smart.embl.de/

7
https://web.expasy.org/protparam/

8
http://cello.life.nctu.edu.tw/

9
https://itol.embl.de/

10
https://meme-suite.org/meme/

11
https://www.ebi.ac.uk/interpro/search/sequence/

12
https://bioinformatics.psb.ugent.be/webtools/plantcare/html/

13
http://plantregmap.gao-lab.org/binding_site_prediction.php

14
https://software.broadinstitute.org/gatk/

## Supplementary material

Supplementary Figure 1 Chromosomal (chr) localization of 96 *BnCDP* genes on *Brassica napus* chromosomes and contigs. The different colors in chromosomes represent gene density, and *BnCDP* genes from different subfamilies are represented in different colors.

Supplementary Figure 2 Number of *cis*-acting regulatory elements in promoters of *BnCDP* genes in *Brassica napus*. The color scale represents amounts from low (blue color) to high (red color).

Supplementary Figure 3 Venn diagrams analysis of the stress-responsive *BnCDP* genes under different treatments. **(A)** Venn diagram shows overlapping responsive genes in *BnCDP* family among different abiotic stresses. **(B)** Venn diagram shows overlapping responsive genes in *BnCDP* family under *S. sclerotiorum* inoculation and four abiotic stress treatments.
