# Supplementary figures and images for: Genome-wide identification and functional analysis of cupin_1 domain-containing members involved in the responses to Sclerotinia sclerotiorum and abiotic stress in Brassica napus
Source: Front Plant Sci. 2022 Aug 1;13:983786. doi: 10.3389/fpls.2022.983786 (PMC9377217; doi:10.3389/fpls.2022.983786)

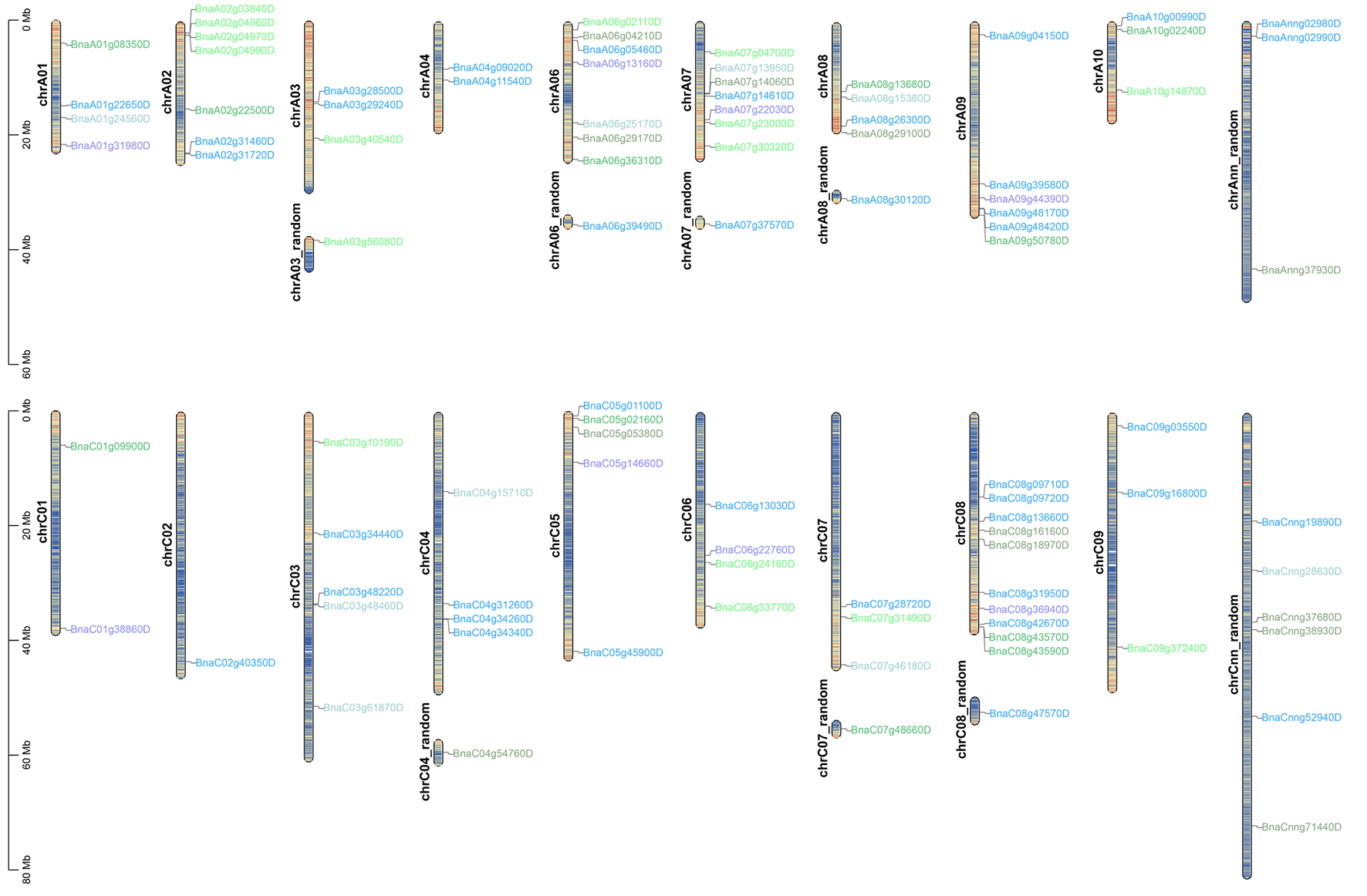

Supplement: Supplementary Figure 1 — Chromosomal (chr) localization of 96 BnCDP genes on Brassica napus chromosomes and contigs. The different colors in chromosomes represent gene density, and BnCDP genes from different subfamilies are represented in different colors. [file Image_1.TIF]

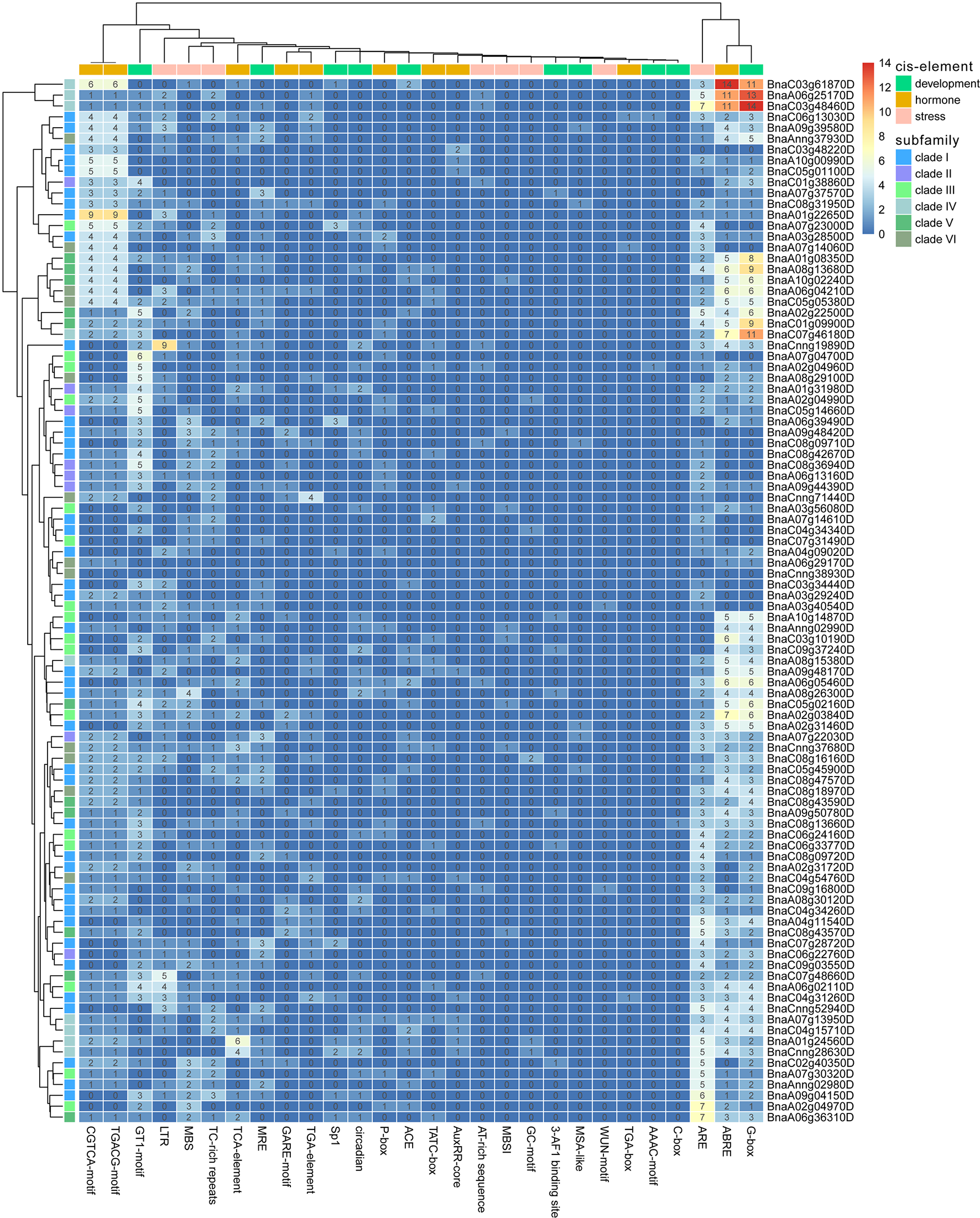

Supplement: Supplementary Figure 2 — Number of cis-acting regulatory elements in promoters of BnCDP genes in Brassica napus. The color scale represents amounts from low (blue color) to high (red color). [file Image_2.TIF]

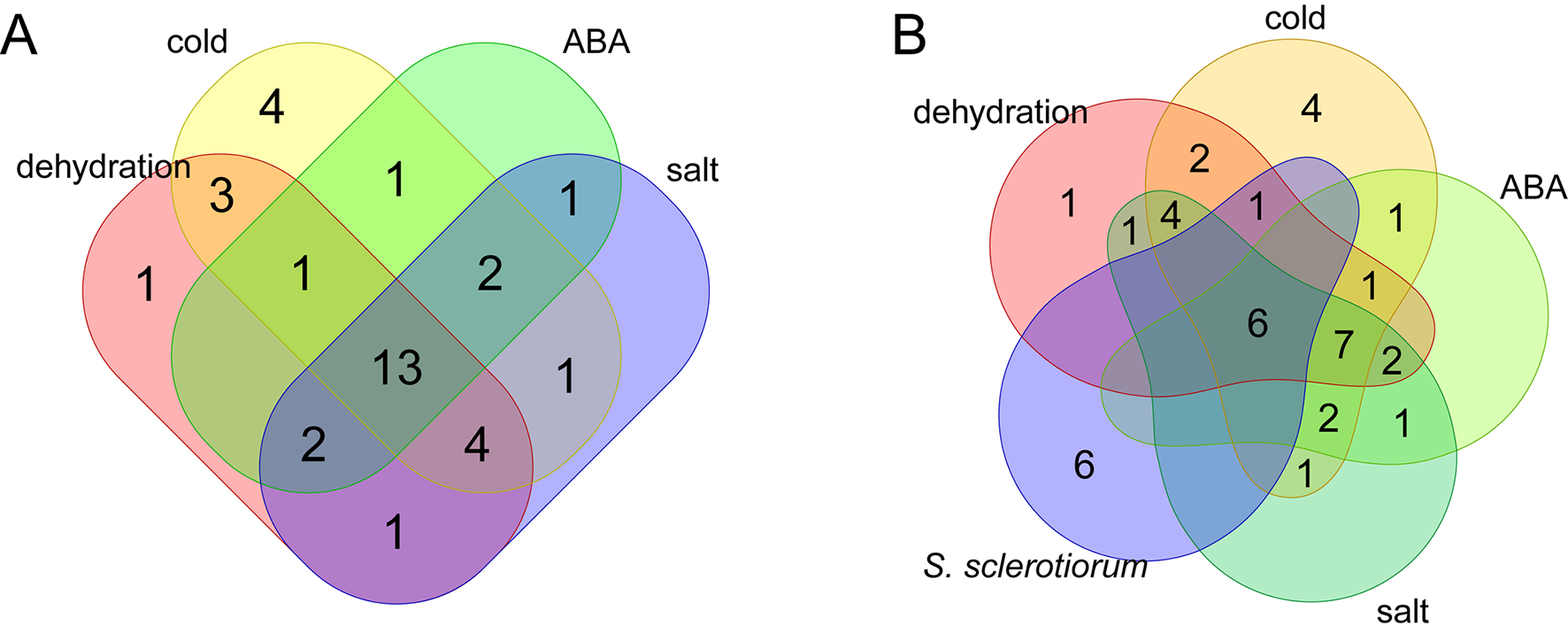

Supplement: Supplementary Figure 3 — Venn diagrams analysis of the stress-responsive BnCDP genes under different treatments. (A) Venn diagram shows overlapping responsive genes in BnCDP family among different abiotic stresses. (B) Venn diagram shows overlapping responsive genes in BnCDP family under S. sclerotiorum inoculation and four abiotic stress treatments. [file Image_3.TIF]
